# T-Cell Heterogeneity in Baseline Tumor Samples: Implications for Early Clinical Trial Design and Analysis

**DOI:** 10.3389/fimmu.2022.760763

**Published:** 2022-04-27

**Authors:** Laura Brennan, Jurriaan Brouwer-Visser, Eveline Nüesch, Maria Karpova, Astrid Heller, Fabien Gaire, Meike Schneider, Bruno Gomes, Konstanty Korski

**Affiliations:** ^1^ Roche Pharma Research and Early Development, Early Biomarker Development Oncology, Roche Innovation Center New York, Little Falls, NJ, United States; ^2^ Roche Innovation Center Basel, Roche Pharma and Early Development, Basel, Switzerland; ^3^ Roche Innovation Center Munich, Roche Pharma and Early Development, Penzberg, Germany

**Keywords:** IHC – immunohistochemistry, CD8 T-cells, Tregs (regulatory T cells), tumor infiltrating lymphocytes (TILs), prior treatment, cancer immunotherapy, intrapatient heterogeneity, multivariate statistics analysis

## Abstract

**Background:**

In early stage clinical trials, changes to levels of tumor infiltrating lymphocytes (TILs) in the tumor microenvironment (TME) are critical biomarkers of the mechanism of action of novel immunotherapies. However, baseline heterogeneity of tumor samples, both between and within patients, and the resultant impact on the validity of clinical trial data is not well defined. Here we identify and quantify the impact of baseline variables on the heterogeneity of FoxP3+ and proliferating CD8+ T-cells levels (MKi67+CD8A+) in the TME both between and within patients for the purpose of informing clinical trial design and analysis.

**Methods:**

We compared levels of FoxP3+ and MKi67+CD8+ cell densities (counts/mm^2^) from >1000 baseline tumor samples from clinical trials and commercially available sources. Using multivariate hierarchical regression techniques, we investigated whether inter-person heterogeneity of activated or regulatory T-cells could be attributed to baseline characteristics including demographics, indication, lesion type, tissue of excision, biopsy method, prior cancer treatment, and tissue type i.e., “fresh” or “archival” status. We also sought to characterize within-patient heterogeneity by lesion type and tissue type.

**Results:**

Prior cancer treatment with hormone therapy or chemotherapy that induces immunogenic cell death may alter the TME. Archival tissue is an unreliable substitute for fresh tissue for determining baseline TIL levels. Baseline and on treatment biopsies should be matched by lesion type to avoid bias.

## Introduction

Cancer immunotherapies (CITs) typically aim to activate immune response and/or reduce immune tolerance to malignancies (1). Incorporating exploratory tissue biomarkers into early phase clinical trials can provide evidence that experimental CITs are modulating the TME as hypothesized and can expand the value of early clinical trial data beyond dose selection and safety assessment (2).

The density of proliferating CD8+ T-cells (MKi67+CD8+) in tumor sections is a commonly used measure of immune activation and is positively associated with a good prognosis across several cancer indications (3–7). CD8+ T-cells include cytotoxic T-cells, which can target and kill tumor cells directly by releasing granules containing perforin and granzyme or *via* cytokines including TNF and IFN-γ (8–10). An established biomarker of immune tolerance is the level of forkhead box transcription factor 3 (FoxP3) in tumors. FoxP3 is a specific marker for regulatory T-cells (Tregs) (11), CD4+ T-cells responsible for depriving cytotoxic T-cells of co-stimulatory signals from cytokines and antigen presenting cells resulting in CD8 T-cell death or dormancy (12). In the context of the tumor microenvironment, high FoxP3 + staining is a biomarker of immune suppression that favors cancer progression (12–14).

Accurate and reliable measurement of these biomarkers can be critical to the success of early phase clinical trials (15). Changes to levels of FoxP3+ and MKi67+CD8+ cell densities between baseline (BL) and on treatment (OT) tissue samples provide an early signal of anti-tumor activity and can validate the hypothesized mechanism of action. Combined with mutation status or RNAseq data, levels of these TILs can help scientists understand the biological pathways underlying resistance or response to experimental CITs. Baseline levels of Tregs or proliferating CD8 T-cells can also be used in retrospective analyses to determine if these biomarkers predict response to therapy or the likelihood of adverse events in order to inform patient selection decisions for late stage trials.

Unfortunately, it is often difficult to draw clear conclusions using early stage biomarker assessments despite the considerable cost and effort to acquire these data. Most often, early stage clinical trials in oncology lack a control group and have insufficient power to demonstrate a meaningful change in biomarkers beyond the variation that would usually be expected over time, the natural progression of disease, or that between patients. Without a control group, clinical scientists are dependent on paired BL and OT samples for biomarker comparison. However, it is rare for all clinical trial subjects to have two usable paired biopsies. The National Cancer Institute reports only about 50% of patients in clinical trials have sufficient paired tissue available for analysis (16) due to patient withdrawal, clinical deterioration, extensive necrosis or fibrosis (17), or insufficient tumor content for IHC (18).

Furthermore, heterogeneity of the TILs in these tumors, both within and between patients, can complicate analysis and cast doubt on the reliability of measurements from a single time point or single tumor lesion. Baseline and OT tumor samples can vary within patients by lesion type (primary v metastatic lesions) or tissue type which may confound treatment-related effects. Baseline tissue type can either be “fresh” tissue acquired days prior to study start or “archival” tissue that was collected earlier in the course of patient treatment and stored after fixation. Variation between patients by indication, demographic group, prior treatment, and lesion type may further confound the interpretation of OT changes or BL levels in ways that are usually unaccounted for.

Given how precious tumor biopsy samples are, it is critical to reduce any ambiguity or bias in biomarker data analysis. Therefore, we identified and quantified the impact of baseline patient and tissue characteristics on the heterogeneity of baseline Treg (FoxP3+) and proliferating CD8+ T-cells levels (MKi67+CD8A+) in the TME, both between and within patients for the purpose of informing clinical trial design and data analysis.

## Materials and Methods

### Study Population

Our tumor dataset includes all baseline tissue samples analyzed by Roche Pharma Research and Early Development in Phase 1 or Phase 2 clinical trials from July 2016 and March 2019 and an additional 132 treatment-naïve, commercially available primary tumors (Invidumed, Hamburg, Germany). Full dataset description can be found on **Table 1**. Details on Invidumed Samples are in **Supplemental Table 1**. Paired analyses were performed only on tumor samples from clinical trials as there were no paired samples in the Individumed samples. Written informed consent was obtained from all subjects and all studies were conducted in accordance with Good Clinical Practice guidelines and the Declaration of Helsinki.

**Table 1:** **(A–C)** Dataset description.

A: Prior Cancer Treatment						
IHC Marker Name:		MKI67+CD8+			FOXP3+	
Variable		N		Mean (cells/mm^2^)	N	Mean (cells/mm^2^)
		1293		*49.5*	1350	*116.6*
**Sample Origin**						
Clinical Trial Subjects		1211		*50.9*	1216	*114.6*
Indivumed Samples		82		*27.7*	134	*134.4*
**Age**						
Over 60		463		*50.0*	566	*124.8*
Under 60		383		*69.3*	408	*115.2*
Unknown		447		*35.8*	376	*121.5*
**Sex**						
Male (ref)		493		*63.9*	574	*127.2*
Female		379		*50.5*	431	*107.8*
Unknown		421		*35.5*	345	*126.3*
**Indication**						
CRC		525		*24.6*	570	*108.2*
NSCLC		102		*91.2*	132	*222.6*
BC		64		*45.8*	111	*81.8*
Other (ref)		602		*64.4*	537	*106.5*
* Bladder Cancer*		63		*26.6*	62	*110.0*
* Esophageal Cancer*		19		*28.7*	18	*138.2*
* Gastric Cancer*		25		*65.7*	26	*90.0*
* Head and Neck Cancer*		40		*112.3*	23	*249.2*
* Hepatocellular Carcinoma*		11		*23.8*	10	*31.6*
* Melanoma*		34		*117.8*	27	*156.0*
* Ovarian Cancer*		57		*52.6*	29	*63.0*
* Pancreatic*		46		*38.4*	48	*135.0*
* Prostate Cancer*		3		*15.7*	12	*24.3*
* Renal Cell Carcinoma*		49		*107.4*	48	*100.0*
* Sarcoma*		27		*19.6*	23	*47.0*
* All Others (less than 10 ea)*		68		*90.2*	60	*105.0*
**B: Tumor Characteristics**						
**IHC Marker Name:**		**MKI67+CD8+**			**FOXP3+**	
**Variable**		**N**		**Mean (cells/mm^2^)**	**N**	**Mean (cells/mm^2^)**
**Tissue of Excision**						
Liver		334		*34.0*	373	*75.9*
Lung		134		*81.1*	170	*184.8*
Lymph Node		133		*106.2*	123	*184.4*
Other (ref)		690		*39.8*	684	*109.6*
**Lesion Type**						
Primary		484		*34.0*	525	*133.7*
All Metastasis (ref)		760		*59.5*	791	*111.3*
* Lymph Node Metastasis*		104		*104.2*	89	*178.3*
* Liver Metastasis*		246		*30.2*	280	*80.4*
* Lung Metastasis*		67		*89.3*	71	*163.4*
Unknown		42		*52.1*	34	*110.5*
**Tissue Type**						
Fresh (ref)		418		*76.3*	485	*124.8*
Archival		578		*39.5*	636	*110.4*
Unknown		297		*42.6*	229	*120.9*
**Biopsy Method**						
Resection (ref)		63		*46.3*	125	*118.1*
Core Needle Biopsy		213		*66.1*	207	*108.2*
Excision Biopsy		6		*20.2*	0	*NA*
Unknown		1011		*48.3*	1018	*115.2*
**C: Prior Cancer Treatment**						
**IHC Marker Name:**		**MKI67+CD8+**			**FOXP3+**	
**Variable**		**N**		**Mean (cells/mm^2^)**	**N**	**Mean (cells/mm^2^)**
**Treatment History**					
Ever		1059		*56.7*	1078	*115.4*
Never (ref)		234		*23.5*	272	*137.2*
Unknown		755		*50.9*	727	*110.0*
**Chemotherapy**						
Never (ref)		263		*27.4*	297	*137.5*
Ever		275		*71.0*	327	*125.8*
* Non-ICD Chemo*		151		*88.8*	137	*183.7*
* ICD Chemo*		124		*45.1*	189	*72.4*
**Radiation**						
Ever		160		*77.6*	183	*136.5*
Never (ref)		378		*36.4*	440	*130.8*
**Hormone Related**						
Ever		27		*21.2*	25	*74.5*
Never (ref)		511		*48.2*	598	*134.2*
**CPI**						
Ever		75		*76.7*	62	*250.9*
Never (ref)		464		*42.9*	561	*120.3*
**All Immunotherapy**						
Ever		112		*75.9*	95	*189.1*
Never (ref)		426		*40.1*	528	*123.3*
**Cytotoxic Antibiotics**						
Ever		58		*71.5*	48	*74.4*
Never (ref)		480		*44.1*	575	*136.2*
**Targeted Therapy**						
Ever		219		*55.0*	261	*130.6*
Never (ref)		319		*42.5*	362	*133.2*

### Immunohistochemistry

All tissue samples were collected, processed and paraffin embedded in the participating pathology labs according to the standardized and approved histopathology protocol. Formalin-fixed paraffin embedded blocks were cut at 4 microns and the IHC staining was performed.

For MKi67 CD8 assay, the RUO Discovery Universal procedure on Discovery Ultra was used. The tissue sections were treated with Cell Conditioner 1 for 64 min and then incubated in primary antibody CD8 (SP239, 1:12.5, Spring Biosciences, for 32 min at 38°C). Bound CD8 antibody was detected with UltraMap anti-rabbit alkaline phosphatase (AP) secondary antibody and Discovery Yellow detection kit (Ventana Medical Systems). Subsequently, after heat denaturation, slides were incubated in primary antibody Ki67 (30–9, RTU, Ventana Medical Systems) for 8 min at 38°C. Bound primary antibody was detected with hapten-linked multimer anti-rabbit hydroquinone (HQ) and anti-HQ horseradish peroxidase secondary antibody, followed by Discovery Purple detection kit (Ventana Medical Systems).

For FoxP3 assay, the XT Optiview DAB IHC v4 procedure on Benchmark XT was used. The tissue sections were treated with Cell Conditioner 1 for 32 min and then incubated in primary antibody FoxP3 (236A-E7, 1:100, Abcam) for 60 min at 37°C and positive staining was detected with OptiView DAB detection kit (Ventana Medical Systems).

All sections were counterstained with hematoxylin II (Ventana Medical Systems) for 8 min, bluing solution for 8 min and then dehydrated and cover-slipped. For all assays, appropriate negative and positive controls were performed.

All slides were scanned with iScan HT scanner (Ventana Medical Systems, Tucson, AZ) and imported into the cloud-based digital pathology Roche proprietary platform (IRIS). Tumor area was annotated by a pathologist to exclude necrotic and/or healthy tissue in the biopsy from analysis. Algorithms for the detection and classification of IHC-stained objects on a whole slide basis were written in MATLAB. Algorithms’ results were assessed and approved by board certified pathologists using standard visualization of the cell detection outputs in IRIS.

### Statistical Methods

We initially explored distributions of Tregs and proliferating CD8 T-cells graphically and prior to modeling, log-transformed measures to meet the assumptions of parametric methods. For our univariate and multivariate regression modeling, to meet the assumption of independent observations, we randomly selected one tumor sample per patient. A correlation matrix was used to test for multicollinearity between variables. We calculated the geometric mean ratio (GMR) of our biomarkers (effect) for each level relative to the reference level of each baseline variable of interest and corresponding 95% confidence intervals based on the restricted maximum likelihood from multivariable linear mixed-effects regression models with Clinical Study as random effect. Invidumed samples were categorized a unique clinical study for this purpose.

For our within-patient analysis we only used clinical trial patient samples as there were no paired samples in the Invidumed tumors. Hypothesis testing of biomarker means in samples paired by tissue type or tumor type was done using paired T-tests of means. Linear correlation of marker levels by time since prior treatment or by tissue type was calculated using Pearson’s correlation. All statistical analyses were run using R version 3.5.2 and all figures were created using TIBCO Spotfire version 10.3.3.

### Variable Parameterization

Our dataset includes 33 indications (**Table 1A**). For our regression modeling, we limited indications to the three most common in our dataset [colorectal cancer (CRC), non-small cell lung cancer (NSCLC), breast cancer (BC)] and grouped the remaining indications as “Other”. Similarly, for tissue of excision, samples from the liver, lung and lymph node (LN) were distinct categories in the model; we defined all other tissue sites as “Other”.

We based our definition of prior treatment category using all known previous lines of cancer therapy at the generic drug name level. We defined chemotherapy (chemo) as any broad acting anti-neoplastic agents, antimetabolites, topoisomerase inhibitors, taxanes, platinum compounds, alkylating agents, and vinca alkaloids. Immunogenic cell death (ICD) chemotherapies are chemotherapies that are known to elicit immunogenic cell death which can trigger adaptive immunity (19) and included: idarubicin, epirubicin, doxorubicin, mitoxantrone, oxaliplatin, bortezomib and cyclophosphamide. Checkpoint Inhibitors (CPI) are defined as any drug specifically targeting either cytotoxic T lymphocyte-associated antigen 4 (CTLA4), programmed cell death protein 1 (PD1) or its ligand (PDL1). Immunotherapies included CPIs plus any other drug targeting the immune system (either stimulating or suppressing) including T-cell bispecific antibodies, anti-CD73 antibodies, anti-CD20 antibodies, OX40 inhibitors, cytokines and dendritic cells. Cytotoxic antibiotics included the drugs bleomycin, cefuroxime, doxorubicin, and/or mitomycin. Hormone therapies (HT) included antiandrogens, aromatase inhibitors, sex hormones, gonadotropin and analogs, somatostatin and analogs. Targeted therapy encompassed all tyrosine kinase inhibitors and monoclonal antibodies. Radiation treatment (rad) was labeled as such in the clinical records. A patient was classified as experienced in these drug categories if they had received a single dose of qualifying drug at any time in the course of their previous cancer therapy.

## Results

### Between Patient Analysis

#### Indication and Prior Treatment With Immunogenic Cell Death Chemotherapy Predictive of FoxP3+ Density

**Figure 1:**
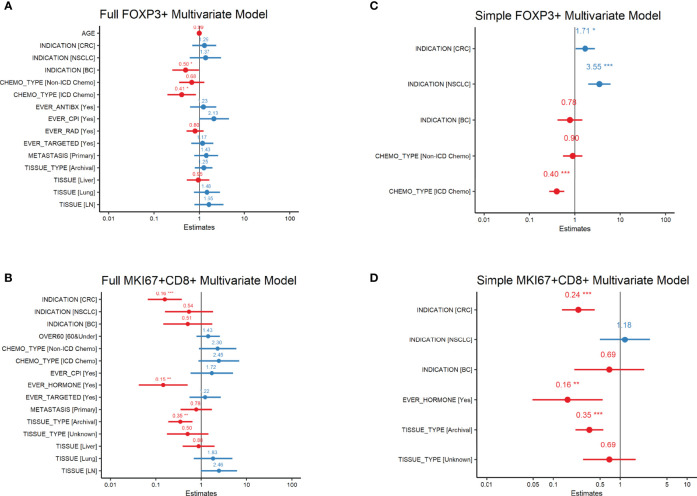
FoxP3+ and MKi67+CD8+ Multivariate Regression Analysis Forest plots of FoxP3+ (regulatory T-cells) or MKi67+CD8+ (proliferating CD8 T-cells) Multivariate Regression Analysis. Estimates are the ratio of geometric means of counts/mm^2^ relative to reference level. All variables in the univariate analysis (**Supplemental Tables 1A, B**) significant at the p=0.1 level or with an effect size of >2 or <0.5 were included in the full multivariate model **(A, B)**. Variables significant at the p ≤ 0.5 level in the full model were then used in the simple model **(C, D)**. P-values are indicated as: ‘***’ 0.001 ‘**’ 0.01 ‘*’ 0.05.

Mean levels of FoxP3+ and MKi67+CD8+ cell counts/mm^2^ by baseline variables are presented in **Tables 1A–C**. In our univariate analysis of FoxP3 densities (**Supplemental Table 2A**), age, prior treatment with immunogenic cell death chemotherapy (ICD-chemo), cytotoxic antibiotics, checkpoint inhibitors, radiation, or targeted therapies, and indication, lesion type (primary tumor vs. metastasis) and tissue of excision were significantly associated with FoxP3+ levels at the p=0.10 significance level and/or had an effect size of >2.0 or <0.5. We incorporated these variables into our full multivariate regression model. In our full multivariate model (**Supplemental Table 2B** and **Figure 1A**) only prior treatment with ICD-chemo and indication remained significantly associated with FoxP3+ levels after controlling for all other variables in the model. Our final parsimonious model (**Supplemental Table 2C** and **Figure 1B**) only included these two variables and explained 32% of the variance of FoxP3+ levels (p-value = 2.78x10^-7^). Prior treatment with ICD-related chemotherapies was associated with 60% lower FoxP3+ cell densities than chemo naïve tumors (GMR=0.40, 95% CI 0.27 to 0.58). FoxP3+ levels in CRC and NSCLC tumors were 1.7 and 3.5 times higher than other indications respectively (CRC 95%CI 1.04 to 2.82, NSCLC 95%CI 2.01 to 6.25).

#### Indication, Tissue Type, and Prior Treatment With Hormone Therapy Predictive of MKi67+CD8+ Cell Density

In our univariate analysis of MKi67CD8+ T-cell densities (**Supplemental Table 3A**) indication, lesion type, tissue of excision, fresh versus archival status and prior treatment with either chemotherapy, CPI, hormone therapy or targeted therapy was associated with MKi67+CD8+ levels at the p=0.10 significance level and/or had an effect size of >2.0 or <0.5. When we included all of these variables into our full multivariate model (**Supplemental Table 3B** and **Figure 1C**), only indication, fresh versus archival tissue status and prior treatment with hormone therapy remained statistically significant. Our final parsimonious model (**Supplemental Table 3C** and **Figure 1D**) included only these three variables and explained 48% of the variance (p=2.41x10^-13^). Tumors from CRC had approximately a quarter of the level of MKi67+CD8+ T-cells compared to other indications (GMR=0.24; 95%CI: 0.135 to 0.42). Archival tissue was more than 60% lower in MKi67+CD8+ T-cells than fresh tissue (GMR =0.35, 95%CI: 0.214 to 0.56) and prior cancer treatment with a hormone therapy was also associated with approximately 84% lower levels of proliferating CD8 T-cells (95%CI: 0.049 to 0.55) compared to tissue from patients who did not receive hormone therapy. We found no meaningful linear relationship between time since last treatment for each treatment category and either biomarker level (**Supplemental Table 4**).

### Within Patient Paired Analysis

#### Density of Proliferating CD8 T-Cells Higher in Metastatic Tissue Compared to Primary Tumors From the Same Patient

We found a statistically significant difference (**Table 2**) in the mean level of MKi67+CD8+ T-cells between primary tumors and paired metastases for any tissue site (p=2.53x10^-5^). Metastases were on average 2.5 times higher in proliferating CD8 T-cells than primary tumors (73.15 counts/mm^2^ vs 26.97 counts/mm^2^). We observed higher MKi67+CD8+ cell counts in metastases regardless of if it was from the liver, lymph node or lung. There was no significant difference in FoxP3+ levels in primary tumors and paired metastases overall (p-value=0.44), or if the paired metastases was limited to the liver, lymph node or lung.

**Table 2:** Paired t-tests by tissue type and lesion type.

IHC Marker	Cell Population	Pairing within Patient	Mean Marker (count/mm2)	N	SD	IQ Range	P-value*	% Pairs Marker Ratio > 2-fold
**FOXP3+**	Regulatory T-cells	distant metastasis	108.62	131	135.30	104.30	0.44	70%
		*primary*	*96.53*	*131*	*108.27*	*130.25*		
		liver metastasis	84.7	70	107.28	70.63	0.42	66%
		*primary*	*87.62*	*70*	*91.33*	*129.53*		
		ln metastasis	121.47	21	128.99	130.90	0.73	78%
		*primary*	*126.37*	*21*	*160.06*	*208.80*		
		lung metastasis	174.95	15	150.04	233.90	0.16	87%
		*primary*	*82.47*	*15*	*93.63*	*65.05*		
		archival	106.67	248	159.58	122.88	0.11	65%
		*fresh*	*100.8*	*258*	*132.20*	*104.38*		
**MKI67+ CD8A+**	Proliferating CD8A+ T-cells	distant metastasis	73.15	108	125.76	49.10	2.50E-05	73%
		*primary*	*26.97*	*108*	*46.72*	*19.95*		
		liver metastasis	52.18	56	89.62	24.30	0.02	70%
		*primary*	*22.77*	*56*	*36.56*	*16.65*		
		ln metastasis	94.58	16	21.51	33.40	0.01	93%
		*primary*	*44.95*	*16*	*22.54*	*29.30*		
		lung metastasis	103.13	13	218.61	83.60	9.70E-04	77%
		*primary*	*11.63*	*13*	*10.45*	*14.80*		
		archival	39.54	206	97.11	28.98	2.10E-03	75%
		*fresh*	*61.17*	*221*	*115.02*	*37.60*		

*Paired T-Test of means.

**Figure 2:**
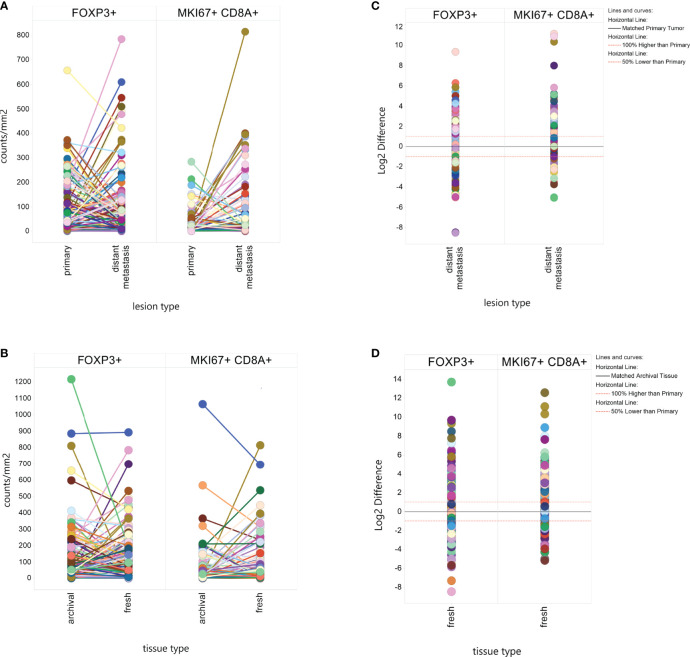
Within Patient Heterogeneity by Lesion Type and Tissue Type. Individual biomarker measurements were plotted by lesion type **(A)** or tissue type **(B)** with line connecting values from the same patient. The difference in Log2 marker level between a distant metastasis and a paired fresh metastasis or between a fresh tissue sample and an archival tissue sample was plotted to calculate what percent of matched pairs displayed a 2-fold (Log2 ≥ |1|) or greater difference by lesion type **(C)** or tissue type **(D)**.

A visual representation of differences in marker levels by lesion type (**Figures 2A–D**), revealed large variation between paired samples. We quantified the variation in a more unbiased method by measuring the Log2 ratio of metastatic tissue levels to primary tumor levels within individuals and then calculated the percent of metastases that had at least a 2-fold difference. The results are presented in **Table 2**. Liver metastases had the lowest within patient-variation, but even then, 70% of MKi67+CD8+ measurements and 66% of FoxP3+ measurements in liver metastases displayed a ≥2-fold difference from their primary pairs.

We found significant differences in mean MKi67+CD8+ levels by tissue type, with fresh tissue on average having higher levels than paired archival tissue (61.17counts/mm^2^ v 39.54 counts/mm^2^, p-value=0.002), but no significant difference in FoxP3+ counts (100.8 in fresh, 106.7 in archival, p=0.11) (**Table 2**). Again, we calculated the percent of paired fresh and archival tissue marker levels having at least a 2-fold difference (**Table 2** and **Figure 2D**). 65% of FoxP3+ measurements and 75% of MKi67+CD8+ measurements had a ≥2-fold density difference between fresh and archival pairs.

#### Poor Correlation Between Fresh and Archival Tissue Pairs Within Patients Even Controlling for Tissue of Excision

**Figure 3:**
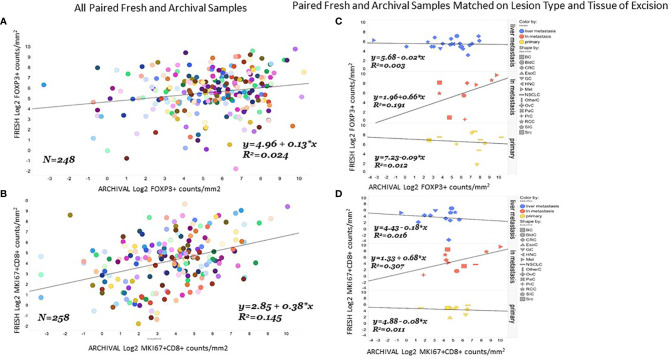
Correlation of FoxP3+ or MKi67CD8+ levels in fresh and archival tissue from the same patient strongest in lymph node metastases. Correlation and linear regression lines were calculated for FoxP3+ **(A)** or MKi67+CD8+ **(B)** levels in all paired archival (X-Axis) and fresh (Y-axis) tissue from the same patient. The same analysis was repeated in paired fresh and archival samples that also matched on tissue of excision and lesion type **(C, D)**.

We found the correlation between fresh and archival tissue pairs to be statistically significant but weak for both Log2 FoxP3+ (**Figure 3A**, R^2 ^= 0.024. β=0.13) and Log2 MKi67+CD8+ (**Figure 3B**, R^2 ^= 0.15, β=0.38). We speculated that some of this discordance might be from differences in the tissue of excision or lesion type between fresh and archival pairs. In order to homogenize our within-patient samples, we did separate analyses of fresh versus archival pairs from the same tissue of excision and lesion type (**Figures 3C, D**). Here we see that only in lymph node metastases is there any relationship in marker levels between fresh and archival pairs (MKi67+CD8+; R^2^ = 0.31, β=0.68. FoxP3+: R^2 ^= 0.19, β=0.66) taken from the same patient.

#### Levels of Proliferating CD8 T-Cells Are Inversely Related to Age of Biopsy in Paired Fresh and Archival Tissue. The Ratio of Proliferating CD8 T-Cells Levels in Fresh Relative to Paired Archival Tissue Increases With Time

**Figure 4:**
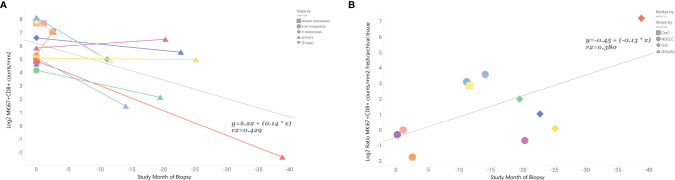
MKi67+CD8+ levels are inversely related to time prior to fresh biopsy in paired fresh and archival samples. Ratio of fresh to archival levels increases with age of archival sample. In 11 paired fresh and archival samples biopsy dates were used to calculate the time archival tissue samples were collected relative to study start and collection of fresh tissue. Fresh tissue is at study month=0. **(A)** Linear correlation of Log2 MKi67+CD8+ cell counts/mm^2^ and study month of biopsy collection. Colored lines connect tissue samples from the same patient. Data points are shaped by lesion type. **(B)** Correlation of Log2 MKi67+CD8+ ratio Fresh/Archival tissue from the same patient. Data points are shaped by patient’s indication. Additional clinical variables are provided in **Supplemental Table 5**.

Finally, we tried to estimate how the time between collection of fresh and archival biopsy —the “age” of the archival sample—influenced the relationship between marker levels in paired fresh and archival samples. We only had biopsy dates for 11 of our fresh/archival pairs and only for MKi67+CD8+ levels. The study month of biopsy for the archival tissue was calculated relative to the date of the paired fresh biopsy (where study month=0) for each individual. Our results (**Figure 4**) show that 43% of the variance in MKi67+CD8+ levels in fresh/archival pairs is explained by the time between collection of biopsies and that the levels of proliferating CD8 T-cells tend to decrease with age of tissue (R^2^ = 0.42, β=0.14, p-value <0.001). We also found that the ratio of MKi67+CD8+ levels between fresh and archival tissue increased with the age of the archival tissue (**Figure 4B**, R^2^ = 0.38, β= **-**0.13).

## Discussion

Our initial aim was to identify which patient characteristics are associated with baseline markers of immune tolerance or activation. After controlling for other potential confounding variables, indication and prior treatment with ICD-chemotherapy remained significant predictors of FoxP3+ levels in tumors.

To the best of our knowledge, this is the first study to demonstrate an association between lower FoxP3+ cell densities in patient tumors and prior ICD-chemotherapy. ICD-related chemotherapies have demonstrated *in vivo* an ability to elicit a specific type of tumor cell death that releases damage-associated molecular patterns (DAMPs) (20, 21). This can trigger the expansion of T-cells specific for tumor antigens (22). While we did not find a relationship between prior ICD-therapy and levels of MKi67+CD8+ cells, the lower levels of FoxP3+ cells observed in ICD-experienced patients may reflect a more immunocompetent phenotype *via* a reduction in immunosuppressive Treg levels. We note that radiotherapy is also a reported ICD inducer (23) but we found no relationship between prior radiation treatment and levels of either marker in our multivariate models [(**Supplemental Table 2**) GMR: 0.71 (95%CI 0.51 to 1.1)]. We hypothesize that systemic ICD-chemotherapy produces a larger and more diverse array of damage associated molecular patterns for dendric cells to recognize than localized radiation therapy does. Future studies should test this experimentally and investigate levels of TILs in tumor samples collected before and after ICD chemotherapy to establish causality.

Our multivariate regression model of MKi67+CD8+ levels revealed an association between indication, tissue type and prior hormone treatment after controlling for all other variables significant in our univariate analysis. Prior hormone therapy was associated with significantly lower levels of proliferating CD8 T-cells (GMR: 0.18) even when controlling for indication. These results are not surprising given the demonstrated anti-proliferative effects of hormone blocking therapies (24). The estrogen receptor (ER) is expressed on T-cells (25) and has been reported to promote T-cell activation and proliferation (26). Expression of IFN-γ by CD8 T-cells is enhanced by estrogen and is critical for CD8 T cell differentiation and response to antigens (27). IL-4 has been shown to stimulate CD8+ cell proliferation in mice (28) and ER-alpha blockers can reduce IL-4 expression by CD8+ T-cells (29). However, these results should be interpreted with caution as the number of hormone therapy experienced patients in our dataset was small (N=27) and our results may reflect ER status among breast cancer patients (30) which we were unable to control for. Future analyses should prospectively investigate the effect of specific hormone therapies on the level of proliferating CD8+ T-cells in the TME.

Our results can help inform patient selection. For example, if we are looking to enrich our study population with low levels of proliferating CD8 T-cells, we may wish to focus on CRC patients or patients who have a history of hormone therapy treatment. Likewise, a clinical trial looking to target patients with high levels of Tregs should consider limiting breast cancer patients or patients who have received ICD-chemo in the past.

Our second aim was to identify how baseline markers of immune exhaustion or activation varied within patients. We found large variation in our biomarker levels between primary tumors and metastatic lesions within the same patient. The variation in FoxP3+ levels was random but MKi67+CD8+ levels tended to be lower in primary tumors compared to metastatic lesions in the same patient. Patient selection efforts should consider this heterogeneity and anticipate that experimental CITs may work differently in primary lesions than in metastases based on their BL levels of TILs. TILs are a localized biomarker; levels in one lesion are not a whole-body reflection of immunogenicity. The TME is dynamic, and our comparison of fresh and archival tissue show that these biomarkers are also not constant over time. Therefore, the use of archival tissue for measuring biomarker levels for patient selection should be avoided as we demonstrated it is a poor reflection of levels in fresh tissue taken at baseline.

Differences between patients with respect to indication or prior treatment with HT or ICD-chemo are unlikely to bias the interpretation of treatment-related effects; indication and treatment history do not change over the course of treatment. However, if availability of follow-up samples is related to indication or treatment history then the analysis of changes to mean biomarker levels over treatment should control for these variables as potential confounders.

The association between fresh versus archival tissue and mean MKi67+CD8+ levels both within and between patients is a potential confounder of treatment related effects. Archival tissue will always be a BL sample and never an OT sample so there is an inherent source of bias. As archival tissue was associated with lower mean levels of MKi67+CD8+, compared to those in fresh baseline samples, failing to control for tissue type will lead to an overestimation of the increase in MKi67+CD8+ with treatment. Our limited data with time between archival and fresh tumor collection indicates the within-patient difference in MKi67+CD8+ levels by tissue type increases with time, but we were unable to identify a clear time threshold for the acceptable collection window for archival tissue. The differences we observed were greater than what would be expected from tissue degradation alone (31, 32) and could be due to tumor progression, increasing tumor mutation burden or intervening cancer therapy. All but 2 of our paired samples in **Figure 4** had 1 or 2 intervening cancer therapies after the archival sample was collected and those 2 patients also had the shortest time between collection of their fresh and archival samples (**Supplemental Table 5**). Our results in **Figure 4** could be biased by differences in lesion type between fresh and archival tissue. The archival tissue is more often from the primary tumor while the fresh tumor was usually from a metastasis. Our paired analysis on lesion types (**Table 2**) showed that metastases tend to be higher in proliferating CD8+ T cells than primary tumors. However, controlling for lesion type (**Figures 3C, D**) did not improve the agreement between fresh and archival tissue in the overall dataset and would not explain the trend we see with age of archival tissue. We found the correlation between paired fresh biopsies from the same patient (**Supplemental Figure 2**) was much stronger than paired fresh and archival patients even without controlling for lesion type. Our data, while limited, indicate that archival tissue collected more than a few weeks before study start would likely be a poor representative of a true baseline sample. Therefore, we recommend that fresh tissue should preferably be collected at enrollment. However, we acknowledge that this is not always practical or even possible for some indications like glioblastoma and requiring fresh tissue for treatment eligibility can be a significant barrier to enrollment, increase the cost of trials, add risk for patients and delay time to treatment (33, 34).

In order to detect a meaningful treatment-related change within individuals, the magnitude of change should always be greater than baseline heterogeneity. Here we see that if baseline and on-treatment samples vary by tissue type or lesion type we could easily observe a doubling of marker levels within patients, unrelated to treatment. To minimize this, if BL and OT samples do vary by tissue or lesion type, the analysis should focus on changes to the mean biomarker levels before and after treatment and not the change within individuals where variation is greater. This is especially true for FoxP3+ levels as there was no statistically significant difference in mean levels by tissue or lesion type.

This study has several strengths. We had a large dataset, with a standardized set of markers measured by standard and accepted methods, well-documented clinical variables including demographics, prior treatment, and biopsy method. Our dataset included paired samples that enabled us to look at differences in levels of TILs both between and within patients. Our tumor samples were representative of samples obtained from clinical trials - heavily pretreated, treatment resistant, advanced cancers - and are ideal for informing clinical development.

Our study was limited by lack of biopsy dates for most archival tissue. Unfortunately, as the archival biopsies were acquired prior to study enrollment this information was not captured with our clinical trial data. We also did not have complete clinical variables for all tissue samples for which we had biomarker levels. Hormone therapy results may be confounded by ER status of breast tissue, which we did not capture in our data. There was not enough racial diversity within our clinical trial sample to examine any effect of race on these marker levels. Finally, this was a cross-sectional analysis, and our results demonstrate associations only and should not be interpreted as causal.

## Conclusions

Here we identified indications and prior cancer treatments that were independent predictors of biomarker levels between patients and may inform patient selection efforts. We also demonstrated that many other variables including age, sex, biopsy type, and most prior cancer therapies (excluding hormone-related and ICD-chemotherapy) do not strongly contribute to the baseline heterogeneity of Treg or proliferating CD8 T-cell levels, and therefore do not need to be controlled for either by design or in statistical analyses of clinical data. Our within-patient analysis highlighted the need for fresh tissue biopsies at baseline matched by lesion type in order to isolate treatment-related modifications to TILs.

Robust biomarker data in early stage clinical trials can support decision-making and inform late stage clinical trial design by demonstrating target engagement, proof of mechanism and investigating subpopulations likely to respond to treatment. The limitations of early clinical trials—small study populations, single arm study design—make efficient design and analysis of paramount importance to maximize the validity of data generated from scarce tissue samples.

## Data Availability Statement

The data analyzed in this study is subject to the following licenses/restrictions: Sharing of individual patient data from clinical trials is restricted by Informed Consent Agreements. Further inquiries can be directed to the corresponding authors. Requests to access these datasets should be directed to bruno.gomes.bg1@roche.com.

## Ethics Statement

Ethical review and approval was not required for the study on human participants in accordance with the local legislation and institutional requirements. The patients/participants provided their written informed consent to participate in this study.

## Author Contributions

LB, JB-V, FG, MS, BG, and KK contributed to the conception and design of the study. LB and EN did the statistical analysis. Figures were made by LB. IHC data was collected and curated by MK and AH. All authors critically revised and approved the final version of the manuscript.

## Funding

Funding was provided by Roche’s Pharma Research and Early Development Department. LB’s postdoc in the Early Biomarker Development Oncology group was funded by the Roche Postdoctoral Fellowship.

## Conflict of Interest

LB, JB-V, EN, MK, AH, FG, MS, BG, and KK are employees of F. Hoffmann-La Roche or were employees at the time this work was completed. This paper does not reference any Roche drug or product.

## Publisher’s Note

All claims expressed in this article are solely those of the authors and do not necessarily represent those of their affiliated organizations, or those of the publisher, the editors and the reviewers. Any product that may be evaluated in this article, or claim that may be made by its manufacturer, is not guaranteed or endorsed by the publisher.

## Supplementary Material

Supplementary Table 1 Invidumed Samples Patient Characteristics. All Invidumed samples were commercially available, untreated primary samples from resected tissue.

Supplementary Table 2 FoxP3+ Univariate and Multivariate Regression Analysis. The association between FoxP3+ cells/mm^2^ in tumor biopsies and each variable of interest was determined in a linear mixed effect model using restricted maximum likelihood estimation **(A)**. Variables with a p-value ≤0.1 or an effect size of >2 or <0.5 were included in the full multivariate model **(B)**. The simple model **(C)** was limited to variables that were significant in the full model at the p ≤ 0.05 level.

Supplementary Table 3 MKi67+CD8+ Univariate and Multivariate Regression Analysis. The association between MKi67+CD8+ cells/mm^2^ in tumor biopsies and each variable of interest was determined in a linear mixed effect model using restricted maximum likelihood estimation **(A)**. Variables with a p-value ≤0.1 or an effect size of >2 or <0.5 were included in the full multivariate model **(B)**. The simple model **(C)** was limited to variables that were significant in the full model at the p ≤ 0.5 level.

Supplementary Table 4 Correlation of Marker Level With Time Since Prior Cancer Therapy.

Supplementary Table 5 Clinical Variable Details for Patients in **Figure 4**.

Supplementary Figure 1 Within Patient Heterogeneity by Specific Lesion Type. Individual biomarker measurements were plotted by specific lesion type with a line connecting values from the same patient. The difference in Log2 marker level between the metastasis and its paired primary lesion was plotted to calculate what percent of lymph node (ln) metastases **(A)**, liver metastases **(B)** or lung metastases **(C)** displayed a 2-fold (Log2 ≥ |1|) or greater difference.
