# Supplementary figures and images for: T-Cell Heterogeneity in Baseline Tumor Samples: Implications for Early Clinical Trial Design and Analysis
Source: Front Immunol. 2022 Apr 27;13:760763. doi: 10.3389/fimmu.2022.760763 (PMC9086966; doi:10.3389/fimmu.2022.760763)

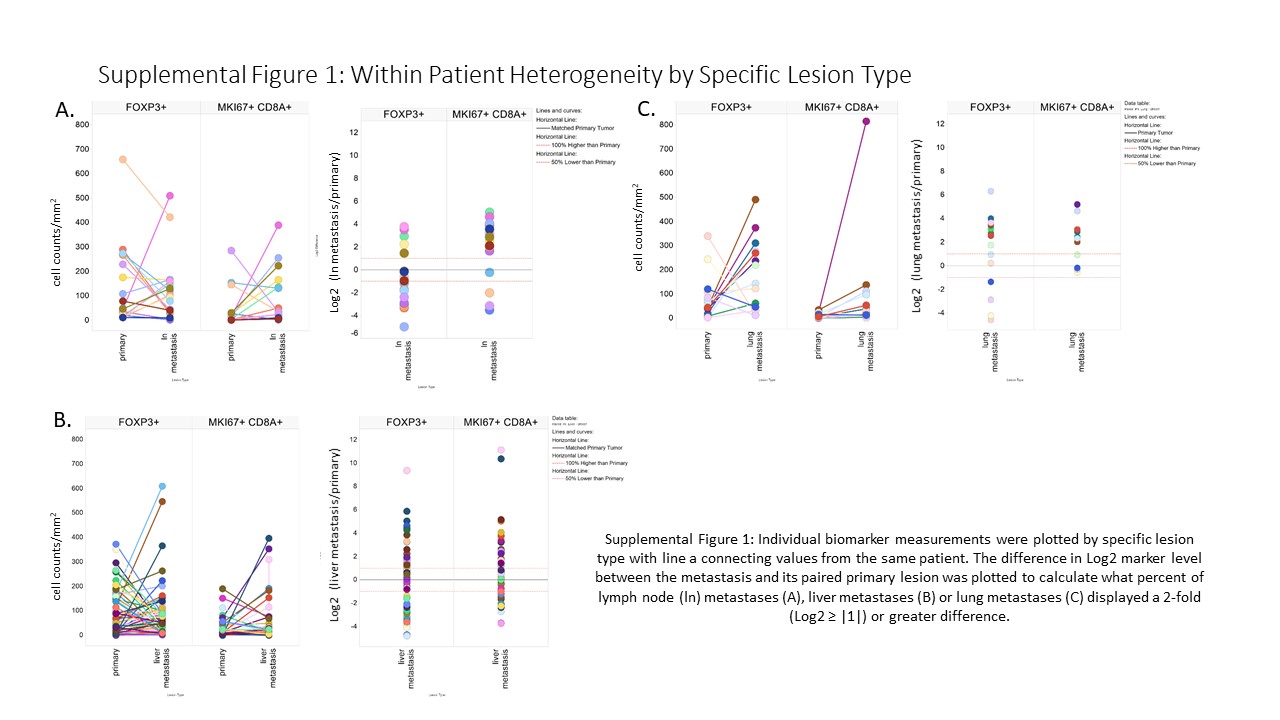

Supplement: Supplementary Figure 1 — Within Patient Heterogeneity by Specific Lesion Type. Individual biomarker measurements were plotted by specific lesion type with a line connecting values from the same patient. The difference in Log2 marker level between the metastasis and its paired primary lesion was plotted to calculate what percent of lymph node (ln) metastases (A), liver metastases (B) or lung metastases (C) displayed a 2-fold (Log2 ≥ |1|) or greater difference. [file Image_1.jpeg]

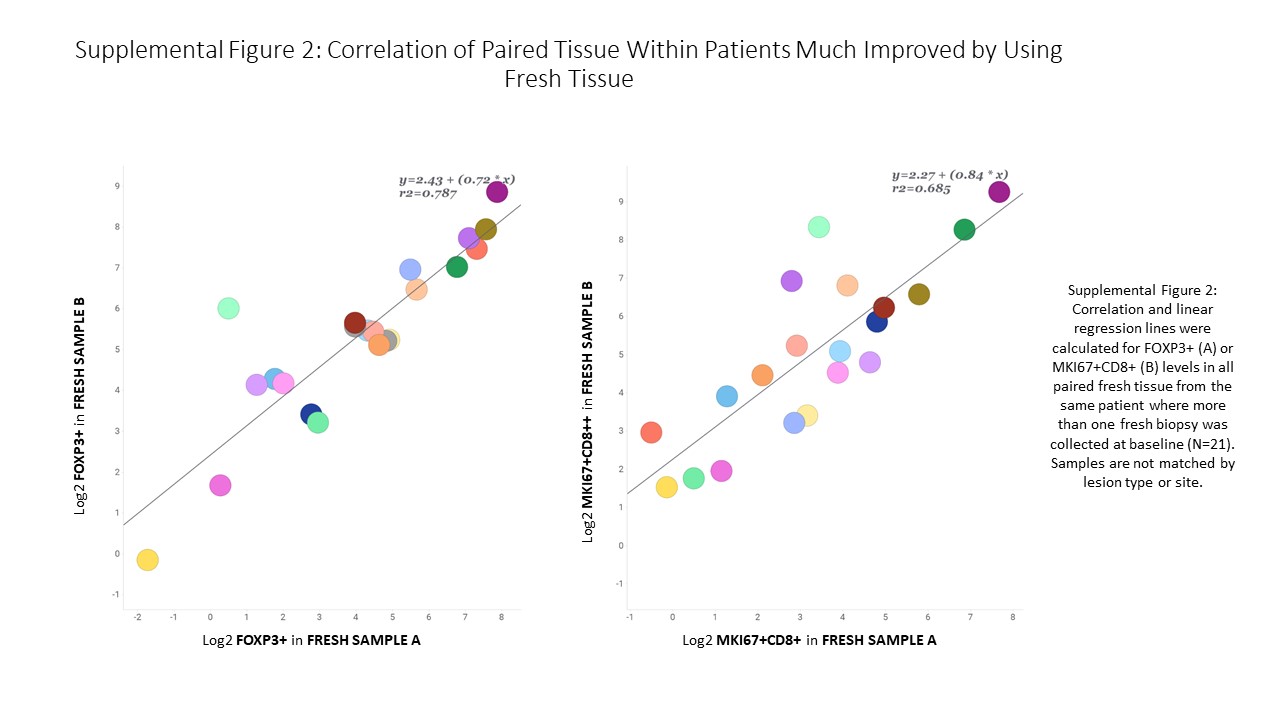

Supplement: Supplementary file 3 [file Image_2.jpeg]
